# Coherent control of Optical limiting in atomic systems

**DOI:** 10.1038/s41598-020-59425-1

**Published:** 2020-02-17

**Authors:** Mohsen Ghaderi Goran Abad, Mahsa Mahdieh, Mohadeseh Veisi, Hamid Nadjari, Mohammad Mahmoudi

**Affiliations:** Department of Physics, University of Zanjan, University Blvd., 45371-38791 Zanjan, Iran

**Keywords:** Nonlinear optics, Magneto-optics

## Abstract

Generation and control of the reverse saturable absorption (RSA) and optical limiting (OL) are investigated in a four-level Y-type quantum system. It is demonstrated that the applied laser fields induce the RSA and it can be coherently controlled by either intensity or frequency of the applied laser fields. The effect of the static magnetic field on the induced RSA is studied and we obtain that it has a constructive role in determining the intensity range in which the OL is established in the system. In addition, we find that the transmission of the suggested optical limiter can be decreased either by increasing the length of the medium or by getting the atomic system denser. Finally, the Z-scan technique is presented to confirm our theoretical results. The proposed scheme can be used in designing the coherent optical limiters with controllable threshold and intensity range of the OL.

## Introduction

Atomic coherence offers a systematic basis for the fundamental concepts of the coherent control. Optical devices are generally based on manipulation of the phase, polarization, propagation direction and also the intensity of the optical light, which can be done by atomic coherence due to the applied laser fields. Intensity manipulation of the light is a major basis of designing all-optical switches^1,2^ and optical limiters^2,3^. Optical limiters have received much attention for the increasing demand in protection of optical components in optically based devices. All optical sensors involved in the optical devices may be vulnerable for their sensitivity to the light intensity. In fact, human eye, sensors and other optical sensitive elements have an intensity threshold above which laser-induced damage happens. In optical limiters, the transmission of the light reduces or even becomes constant for the input intensities higher than the threshold intensity. Thus, the presence of optical limiters to restrict the intensity of the incident laser beam is completely requisite prior to the sensors and direct viewing devices. Optical limiters protect the sensors from the damages due to the higher intensity laser pulse by extending their intensity range to operate under rougher conditions.

Various techniques, i.e., two photon absorption^4–6^, nonlinear scattering^7,8^ have been reported for generating the optical limiting (OL). The basic mechanism to establish the OL is the reverse saturable absorption (RSA)^9–11^ in which, unlike the saturable absorption (SA), the absorption increases by increasing the incident intensity. In general, the RSA can occur when the absorption of the excited state is large compared to the absorption of the ground state. On the contrary, SA is the dominant phenomenon in the system. Note that in SA materials, the absorption reduces with increasing the intensity due to depletion of the ground state, leading the materials to be more transparent. The RSA and OL have been observed in numerous compounds such as organic materials^3,12^, *C*_60_ solution^13,14^, complicated molecular structure^15–17^ and semiconductors^18^. Different investigations on the various molecules have demonstrated that the OL behavior is dependent on the density and concentration of the molecules^19,20^, so that Azzam *et al*. suggested thinner films with larger density in OLs device engineering^20^. Moreover, dependence of OL behavior on light wavelength has been previously shown^21,22^. OL features of metal nanowires have been widely investigated because of their applications^22–26^. Pan *et al*.^22^ have explored various metal nanowires and showed that the OL threshold is different for different metal nanowires. Note that all the materials used for generating OL in the previous works are nonlinear materials, which naturally show the RSA. Moreover, for protecting the various optical devices with different OL threshold, one should change the material or its concentration. Here, we induce the nonlinear properties to the medium by applying the laser fields. It leads to design a simpler optical limiter, which the optical limiting properties can be coherently controlled by the applied fields.

In this paper, unlike the previous reported works, we introduce a coherently controllable optical limiter using the atomic systems in a four-level Y-type configuration. It is demonstrated that the RSA is coherently induced by the laser fields and the conditions are provided for preparing the OL in the system. It is shown that all characteristics of the induced OL such as the intensity range, the threshold intensity and the transmission can be controlled by either intensity or frequency of the laser fields. Moreover, the effect of the static magnetic field on the OL is studied and it is illustrated that the RSA and the OL regions are extended by increasing the magnitude of the static magnetic field. In addition, we show that the transmission of the suggested optical limiter decreases either by increasing the length of the medium or by getting the atomic medium denser. Finally, the Z-scan technique is presented to confirm our theoretical results.

## Model and Equations

**Figure 1:**
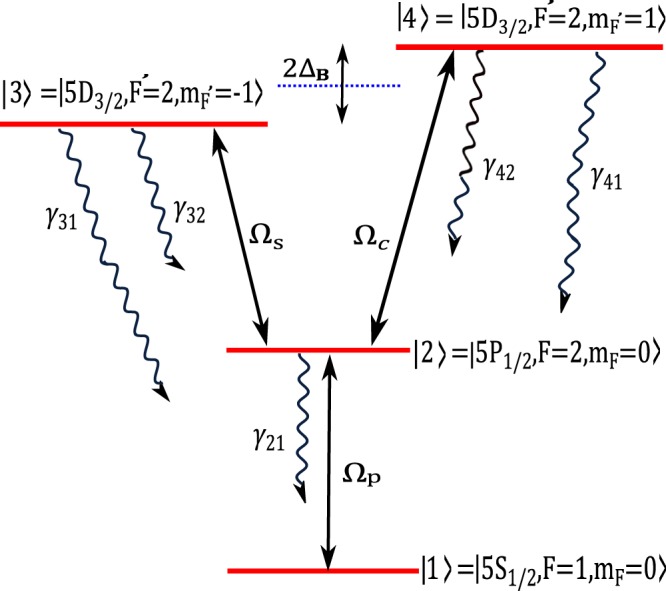
A schematic of a four-level Y-type quantum system driven by a weak probe field and two coupling fields with Rabi frequencies Ω_*p*_, Ω_*s*_ and Ω_*c*_ respectively.

The proposed realistic atomic system is a four-level Y-type quantum system, which can be established in 5*S*_1∕2_, 5*P*_1∕2_ and 5*D*_3∕2_ lines of ^87^Rb atoms as shown in Fig. 1. Two states $| 1\rangle =| 5{S}_{1/2},(F=1,{m}_{F}=0)\rangle$ and $| 2\rangle =| 5{P}_{1/2},(F=2,{m}_{F}=0)\rangle$, separated by 377 THz, are defined as ground state and intermediate state. Two degenerate states $| 3\rangle =| 5{D}_{3/2},({F}^{{\prime} }=2,{m}_{{F}^{{\prime} }}=-1)\rangle$ and $| 4\rangle =| 5{D}_{3/2},({F}^{{\prime} }=2,{m}_{{F}^{{\prime} }}=1)\rangle$ are chosen as the higher excited states. Here, *F* and ${F}^{{\prime} }$ are the quantum numbers of the total angular momentum and ${m}_{F({F}^{{\prime} })}$ denotes magnetic quantum number of the corresponding states. A weak linearly probe field, $\overrightarrow{{E}_{p}}(z,t)={\overrightarrow{\varepsilon }}_{p}(z){e}^{-i({\omega }_{p}t-{k}_{p}z)}+c.c.$, with wave vector *k*_*p*_ and polarization in $\widehat{x}$ direction drives the transition $| 1\rangle \leftrightarrow | 2\rangle$ with the Rabi frequency ${\Omega }_{p}={\overrightarrow{\mu }}_{21}.\overrightarrow{{\varepsilon }_{p}}/\hslash$. The transition $| 2\rangle \leftrightarrow | 3\rangle$ ($| 2\rangle \leftrightarrow | 4\rangle$) is coupled by the strong left (right) circularly polarized coupling field with the Rabi frequency ${\Omega }_{s}={\overrightarrow{\mu }}_{32}.{\widehat{\varepsilon }}_{-}{E}_{s}/\hslash$ (${\Omega }_{c}={\overrightarrow{\mu }}_{42}.{\widehat{\varepsilon }}_{+}{E}_{c}/\hslash$). *ε*_*p*_ and *E*_*i*_(*i* = *s*, *c*) are the amplitude of the probe and coupling fields, respectively. *ε*_±_ stands for the polarization unit vectors of the coupling fields. A static magnetic field is also employed to remove the degeneracy of the higher excited states $| 3\rangle$ and $| 4\rangle$ by 2*ℏ*Δ_*B*_ = 2*m*_*F*_*g*_*s*_*μ*_*B*_*B* where *μ*_*B*_ is Bohr magneton and *g*_*s*_ is Landé factor. This system can make it possible that the intermediate state $| 2\rangle$ to be depleted by applying two strong coupling fields to the higher excited states in the presence of a static magnetic field. Thus the necessary condition is provided for the RSA.

The Hamiltonian of the considered system in the dipole and rotating wave approximations can be written as

1 $${V}_{I}=-\,\hslash ({\Omega }_{p}^{* }{e}^{-i{\Delta }_{p}t}| 2\rangle | 1\rangle +{\Omega }_{s}^{* }{e}^{-i({\Delta }_{s}+{\Delta }_{B})t}| 3\rangle | 2\rangle +{\Omega }_{c}^{* }{e}^{-i({\Delta }_{c}-{\Delta }_{B})t}| 4\rangle | 2\rangle )+h.c.,$$

where Δ_*p*_ = *ω*_*p*_ − *ω*_21_, Δ_*s*_ = *ω*_*s*_ − *ω*_32_ and Δ_*c*_ = *ω*_*c*_ − *ω*_42_ are the detunings. *ω*_*p*_, *ω*_*s*_ and *ω*_*c*_ are the frequencies of the probe and coupling fields, respectively. Also, *ω*_21_, *ω*_32_ and *ω*_42_ are the central frequencies of the corresponding transitions. The density matrix equations of motion can be written as follows

2 $$\begin{array}{lll}{\dot{\rho }}_{11} & = & i{\Omega }_{p}{\rho }_{21}-i{\Omega }_{p}^{* }{\rho }_{12}+{\gamma }_{21}{\rho }_{22}+{\gamma }_{31}{\rho }_{33}+{\gamma }_{41}{\rho }_{44},\\ {\dot{\rho }}_{33} & = & i{\Omega }_{s}^{* }{\rho }_{23}-i{\Omega }_{s}{\rho }_{32}-{\Gamma }_{1}{\rho }_{33},\\ {\dot{\rho }}_{44} & = & i{\Omega }_{c}^{* }{\rho }_{24}-i{\Omega }_{c}{\rho }_{42}-{\Gamma }_{2}{\rho }_{44},\\ {\dot{\rho }}_{21} & = & i{\Omega }_{p}^{* }({\rho }_{11}-{\rho }_{22})+i{\Omega }_{s}{\rho }_{31}+i{\Omega }_{c}{\rho }_{41}-[\frac{{\gamma }_{21}}{2}-i{\Delta }_{p}]{\rho }_{21},\\ {\dot{\rho }}_{31} & = & i{\Omega }_{s}^{* }{\rho }_{21}-i{\Omega }_{p}^{* }{\rho }_{32}-[\frac{{\Gamma }_{1}}{2}-i({\Delta }_{s}+{\Delta }_{B}+{\Delta }_{p})]{\rho }_{31},\\ {\dot{\rho }}_{32} & = & i{\Omega }_{s}^{* }({\rho }_{22}-{\rho }_{33})-i{\Omega }_{p}{\rho }_{31}-i{\Omega }_{c}^{* }{\rho }_{34}-[\frac{{\Gamma }_{1}+{\gamma }_{21}}{2}-i({\Delta }_{s}+{\Delta }_{B})]{\rho }_{32},\\ {\dot{\rho }}_{41} & = & i{\Omega }_{c}^{* }{\rho }_{21}-i{\Omega }_{p}^{* }{\rho }_{42}-[\frac{{\Gamma }_{2}}{2}-i({\Delta }_{c}+{\Delta }_{p}-{\Delta }_{B})]{\rho }_{41},\\ {\dot{\rho }}_{42} & = & i{\Omega }_{c}^{* }({\rho }_{22}-{\rho }_{44})-i{\Omega }_{p}{\rho }_{41}-i{\Omega }_{s}^{* }{\rho }_{43}-[\frac{{\Gamma }_{2}+{\gamma }_{21}}{2}-i({\Delta }_{c}-{\Delta }_{B})]{\rho }_{42},\\ {\dot{\rho }}_{43} & = & i{\Omega }_{c}^{* }{\rho }_{23}-i{\Omega }_{s}{\rho }_{42}-[\frac{{\Gamma }_{1}+{\Gamma }_{2}}{2}+i({\Delta }_{s}-{\Delta }_{c}+2{\Delta }_{B})]{\rho }_{43},\\ {\dot{\rho }}_{22} & = & -({\dot{\rho }}_{11}+{\dot{\rho }}_{33}+{\dot{\rho }}_{44}),\end{array}$$

where Γ_1_ = *γ*_31_ + *γ*_32_ and Γ_2_ = *γ*_41_ + *γ*_42_. The parameter *γ*_*i*1_(*γ*_*i*2_)(*i* = 3, 4) denotes the spontaneous decay rate from the excited state $| i\rangle$ to the lower states $| 1\rangle$ ($| 2\rangle$). The polarization vector in the atomic medium is given by

3 $$\overrightarrow{P}(z,t)={\chi }_{p}\overrightarrow{{\varepsilon }_{p}}{e}^{-i({\omega }_{p}t-{k}_{p}z)}+c.c.$$

Here, *χ*_*p*_ is the susceptibility representing the response of the medium to the probe field.

Let us now solve the wave equation for the probe field, which can be written as

4 $${\nabla }^{2}{\overrightarrow{E}}_{p}-{\mu }_{0}{\epsilon }_{0}\frac{{\partial }^{2}{\overrightarrow{E}}_{p}}{\partial {t}^{2}}-{\mu }_{0}\frac{{\partial }^{2}\overrightarrow{P}}{\partial {t}^{2}}=0.$$

Inserting equation 3 into equation 4 and using slowly varying approximation, we simplify the equation 4 as

5 $$\frac{\partial {\varepsilon }_{p}}{\partial z}=i2\pi {\omega }_{p}{({\mu }_{0}{\epsilon }_{0})}^{1/2}{\varepsilon }_{p}{\chi }_{p}.$$

Thus, the solution of the equation 4 by substituting $c=1/\sqrt{{\mu }_{0}{\epsilon }_{0}}$ and *k*_*p*_ = *ω*_*p*_/*c* leads the output probe field amplitude to become as

6 $${\varepsilon }_{p}(z=l)={\varepsilon }_{p}(0){e}^{i2\pi {k}_{p}l{\chi }_{p}}.$$

*χ*_*p*_ can be related to the probe transition coherence *ρ*_21_ defined as

7 $${\chi }_{p}=\frac{n{\mu }_{21}^{2}{\rho }_{21}}{\hslash {\Omega }_{p}},$$

where *n* is density of atoms and *ρ*_21_ is calculated from equation (2). Therefore, the equation 6 reduces to

8 $${\varepsilon }_{p}(z=l)={\varepsilon }_{p}(0){e}^{i\frac{\alpha l{\rho }_{21}\gamma }{2{\Omega }_{p}}},$$

where $\alpha l=4\pi n{\mu }_{21}^{2}{k}_{p}l/\hslash \gamma$ is the resonant absorption. By introducing the normalized susceptibility *S*_*p*_ = *ρ*_21_*γ*/*Ω*_*p*_, the output probe field amplitude takes the form

9 $${\varepsilon }_{p}(z=l)={\varepsilon }_{p}(0){e}^{i\frac{\alpha l}{2}{S}_{p}}.$$

Finally, the normalized transmission of the probe field is given by

10 $$T=\frac{| {\varepsilon }_{p}(z=l){| }^{2}}{| {\varepsilon }_{p}(0){| }^{2}}={e}^{-\alpha lIm[{S}_{p}]}.$$

The normalized susceptibility *S*_*p*_ is clearly a complex quantity that its imaginary part stands for the absorption of the probe field. The intensity region in which the imaginary part of *S*_*p*_ increases with the increase of the input intensity denotes the RSA region. Equation 10 displays the transmission behavior of the light, which is going to be based on the study of the OL properties of the quantum system.

The Z-scan technique is widely used to study the nonlinear refractive index^27^ as well as the OL properties of various materials^21^. In experiment, a Z-scan setup includes a laser field with a transverse Gaussian profile focused by using a lens. The sample is then moved along the propagation direction of the focused Gaussian field. It is clear that the sample experiences maximum intensity at the focal point (*z* = 0), which gradually decreases in either direction from the focus. The Z-scan technique shows the transmission based on the scanning of the sample position relative to the focal plane of the lens. The incident probe field is a Gaussian laser field with the Rabi frequency Ω_*p*_

11 $${\Omega }_{p}(z,r,t=0)={\Omega }_{p0}\frac{{w}_{0}^{2}}{{w}^{2}(z)}exp[-(\frac{2{r}^{2}}{{w}^{2}(z)})],$$

where Ω_*p*0_ is the probe Rabi frequency at the focal point (beam waist), *w*_0_ = 0.1 *mm* is the beam waist radius at focus, $w(z)={w}_{0}{[1+{(z/{z}_{0})}^{2}]}^{1/2}$ is the beam radius at *z* (the distance of the sample from the focal point) and ${z}_{0}=\pi {w}_{0}^{2}/\lambda$ is the diffraction length of the beam. It should be noted that the Z-scan measurements in our work are carried out for the probe field at 800 *nm* wavelength corresponding to the transition 5*S*_1∕2_ ↔ 5*P*_1∕2_.

With calculations of equation 11 numerically for a pulsed Gaussian beam, normalized transmission as a function of position can be obtained as

12 $$T(z)=\frac{4}{{w}^{2}}{\int }_{0}^{\infty }{\rm{r}}Texp[-(\frac{2{r}^{2}}{{w}^{2}(z)})]dr.$$

## Results and Discussion

**Figure 2:**
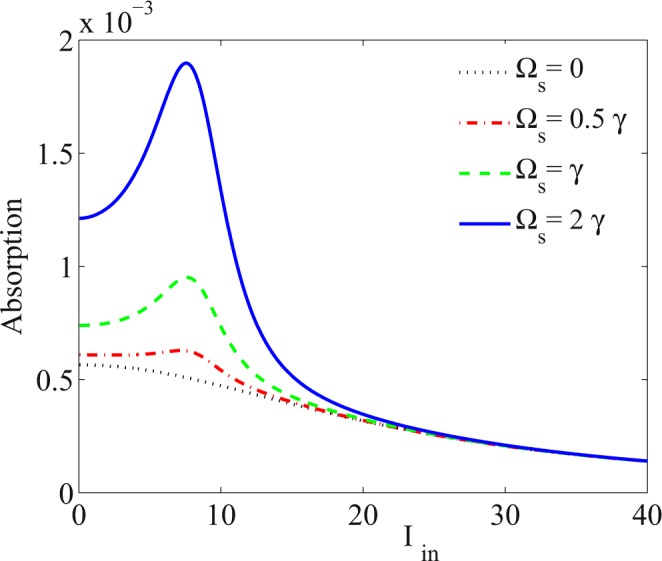
Absorption of the probe field versus intensity of the input probe field for different values of Ω_*s*_. The used parameters are Ω_*p*_ = 0.01*γ*, Ω_*c*_ = 65*γ*, Δ_*c*_ = 100*γ*, Δ_*p*_ = 1.5*γ*, Δ_*s*_ = 0, Δ_*B*_ = 0, Ω_*s*_ = 0 (dotted), Ω_*s*_ = 0.5*γ* (dot-dashed), Ω_*s*_ = *γ* (dashed) and Ω_*s*_ = 2*γ* (solid).

Here, we are going to present our numerical results describing the absorption behavior of the system. We are interested in investigating the SA and RSA regions in the system to provide the appropriate conditions for inducing the OL. All the parameters are scaled by *γ*, which is 2*π* × 5.75 MHz for the transition 5*S*_1∕2_ ↔ 5*P*_1∕2_ of ^87^Rb atoms. Figure 2 shows the absorption of the probe field versus the incident intensity of the probe field for different values of the Ω_*s*_. The dotted line is for Ω_*s*_ = 0, the dot-dashed line for Ω_*s*_ = 0.5*γ*, the dashed line for Ω_*s*_ = *γ* and the solid line for Ω_*s*_ = 2*γ*. The other used parameters are Ω_*p*_ = 0.01*γ*, Ω_*c*_ = 65*γ*, Δ_*c*_ = 100*γ*, Δ_*p*_ = 1.5*γ*, Δ_*s*_ = 0 and Δ_*B*_ = 0. It is seen that when the coupling field Ω_*s*_ is switched off, the absorption of the probe field decreases by increasing the intensity of the input probe field and the SA is dominant in the absence of the Ω_*s*_. By switching on the Ω_*s*_, the RSA is induced and a peak is generated in the absorption of the probe field, leading to separate the RSA and SA regions. Moreover, the absorption peak enhances by increasing the Ω_*s*_, so it allows us to control the RSA phenomenon. Generally, in the RSA region (left side of the peak), the absorption increases by growing the intensity of the input laser field. An investigation on Fig. 2 shows that the RSA can switch to the SA for the intense input laser field.

**Figure 3:**
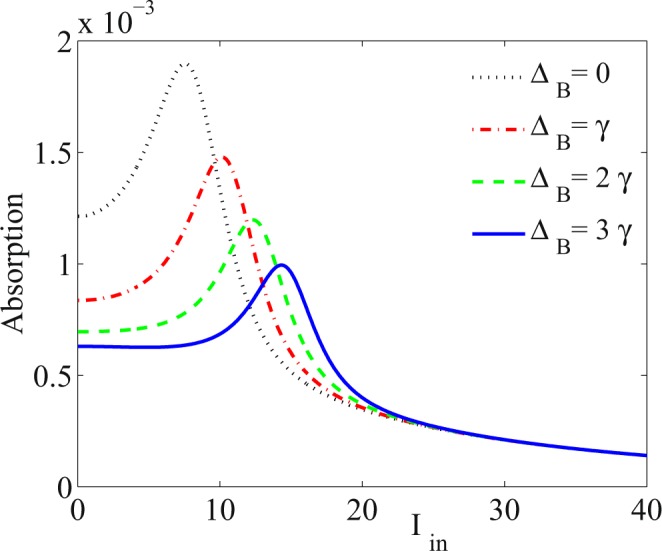
Absorption behavior of the probe field versus intensity of the input probe field for different values of Δ_*B*_. The taken parameters are Ω_*p*_ = 0.01*γ*, Ω_*c*_ = 65*γ*, Δ_*c*_ = 100*γ*, Δ_*p*_ = 1.5*γ*, Δ_*s*_ = 0, Ω_*s*_ = 2*γ*, Δ_*B*_ = 0 (dotted), Δ_*B*_ = *γ* (dot-dashed), Δ_*B*_ = 2*γ* (dashed) and Δ_*B*_ = 3*γ* (solid) corresponding to the static magnetic field *B* = 0, *B* = 2*G*, *B* = 4.3*G* and *B* = 6.4*G*, respectively.

The extension of the RSA with respect to the SA region is another scenario that we can do by applying the static magnetic field. After inducing the RSA in the system, it is important that the system maintains the RSA behavior in wider range of the intensity of the input field. The constructive role of the static magnetic field in the RSA is presented in Fig. 3 for Δ_*B*_ = 0 (dotted), Δ_*B*_ = *γ* (dot-dashed), Δ_*B*_ = 2*γ* (dashed) and Δ_*B*_ = 3*γ* (solid) corresponding to the static magnetic field *B* = 0, *B* = 2*G*, *B* = 4.3*G* and *B* = 6.4*G*, respectively. The other taken parameters are Ω_*p*_ = 0.01*γ*, Ω_*c*_ = 65*γ*, Δ_*c*_ = 100*γ*, Δ_*p*_ = 1.5*γ*, Δ_*s*_ = 0 and Ω_*s*_ = 2*γ*. We result that the RSA region is extended by increasing the magnitude of the static magnetic field and the RSA is established in a larger range of the input probe field intensity, which promises the extension of the OL range.

**Figure 4:**
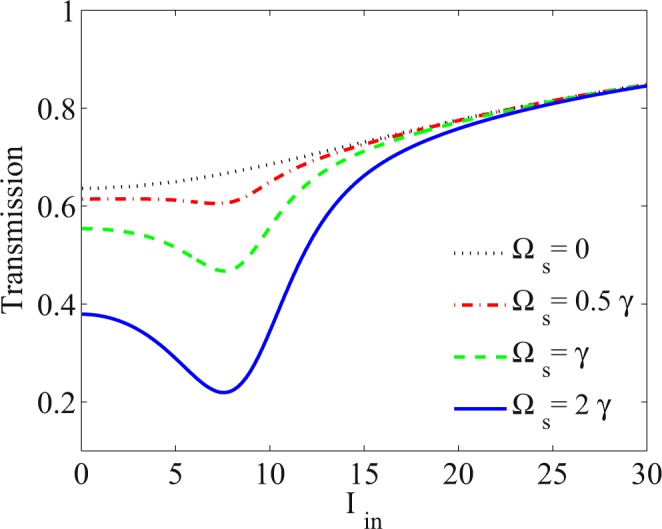
Transmission of the probe field versus intensity of the input probe field for Ω_*s*_ = 0 (dotted), Ω_*s*_ = 0.5*γ* (dot-dashed), Ω_*s*_ = *γ* (dashed) and Ω_*s*_ = 2*γ* (solid). The other used parameters are the same used in Fig. 2 accompanied by *αl* = 800*γ*.

In the following, we investigate the effect of the coupling field, Ω_*s*_, on the transmission of the probe field. In Fig. 4, the transmission of the probe field versus the intensity of the input probe field is shown for *αl* = 800*γ*, Ω_*s*_ = 0 (dotted), Ω_*s*_ = 0.5*γ* (dot-dashed), Ω_*s*_ = *γ* (dashed) and Ω_*s*_ = 2*γ* (solid). The other used parameters are those taken in Fig. 2. Figure 4 shows that in the absence of the coupling field, the transmission of the probe field grows with the increase of the input probe field intensity going the system toward transparency. Thus, the system cannot be used as an optical limiter. As proved in Fig. 2, the RSA was induced and intensified by increasing the Ω_*s*_. In the RSA domain, the transmission of the probe field keeps constant or even reduces wity increasing the coupling field intensity. Hence, it is demonstrated that the OL is coherently induced and controlled in the system. In addition, a bird’s eye view of Fig. 4 reveals that the increase of the Ω_*s*_ can lead to decrease the threshold of the OL induced in the system.

**Figure 5:**
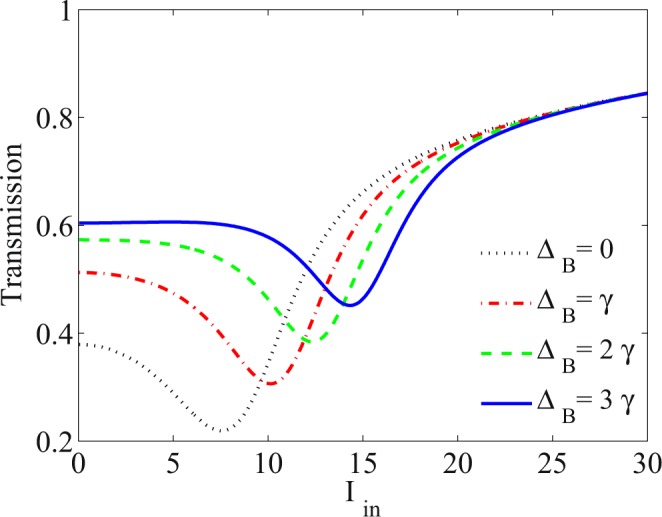
Effect of the static magnetic field as Δ_*B*_ = 0 (dotted), Δ_*B*_ = *γ* (dot-dashed), Δ_*B*_ = 2*γ* (dashed) and Δ_*B*_ = 3*γ* (solid) corresponding to the static magnetic field *B* = 0, *B* = 2 *G*, *B* = 4.3 *G* and *B* = 6.4 *G*, respectively, on the transmission of the probe field versus input intensity of the probe field. The other parameters used are the same as those in Fig. 3 accompanied by *αl* = 800*γ*.

One of the important features that distinguish an optical limiter from the rest is the intensity range in which the optical limiter operates. Here, we show that the applying the static magnetic field can extend the OL range. Figure 5 depicts the transmission of the probe field versus input intensity for different values of the static magnetic field. The taken parameters are the same used in Fig. 3. Figure 5 shows that the OL range can be controlled by the static magnetic field. It is seen that in the absence of the static magnetic field, the OL is even established in a small range of the input intensity. Thus, in our suggested atomic optical limiter, applying the static magnetic field makes the optical devices and sensors safe from damages in a larger range of input intensity.

**Figure 6:**
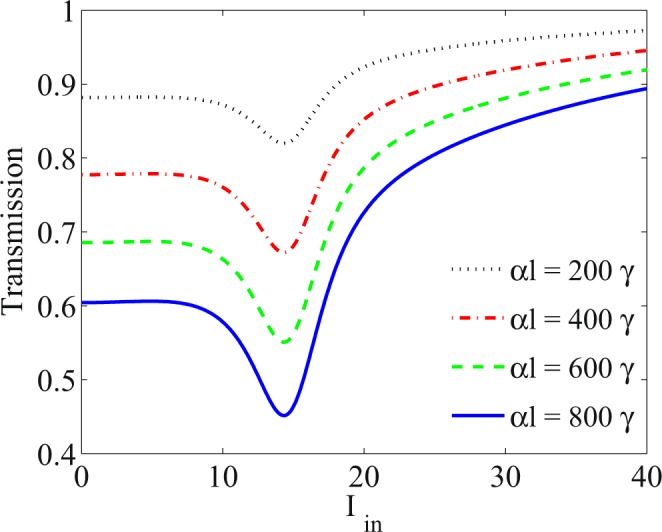
Transmission of the probe field versus the input intensity of the probe field for different values of the resonance absorption *αl* = 200*γ* (dotted), *αl* = 400*γ* (dot-dashed), *αl* = 600*γ* (dashed) and *αl* = 800*γ* (solid). The other taken parameters are Ω_*p*_ = 0.01*γ*, Ω_*c*_ = 65*γ*, Δ_*c*_ = 100*γ*, Δ_*p*_ = 1.5*γ*, Δ_*s*_ = 0, Ω_*s*_ = 2*γ* and Δ_*B*_ = 3*γ*.

Control of the intensity of the transmission is another advantage of the suggested optical limiter. In Fig. 6, the effect of the resonant absorption, *αl*, is studied on the transmission of the probe field plotted versus the intensity of the input probe field. Resonance absorption is directly related to the length of the medium and density of atoms. It is observed that by increasing *αl*, the transmission decreases with the same OL thresholds. Decrease of the transmission makes it possible that the presented optical limiter can be set to use in optical devices, which need the optical limiters with lower transmissions.

**Figure 7:**
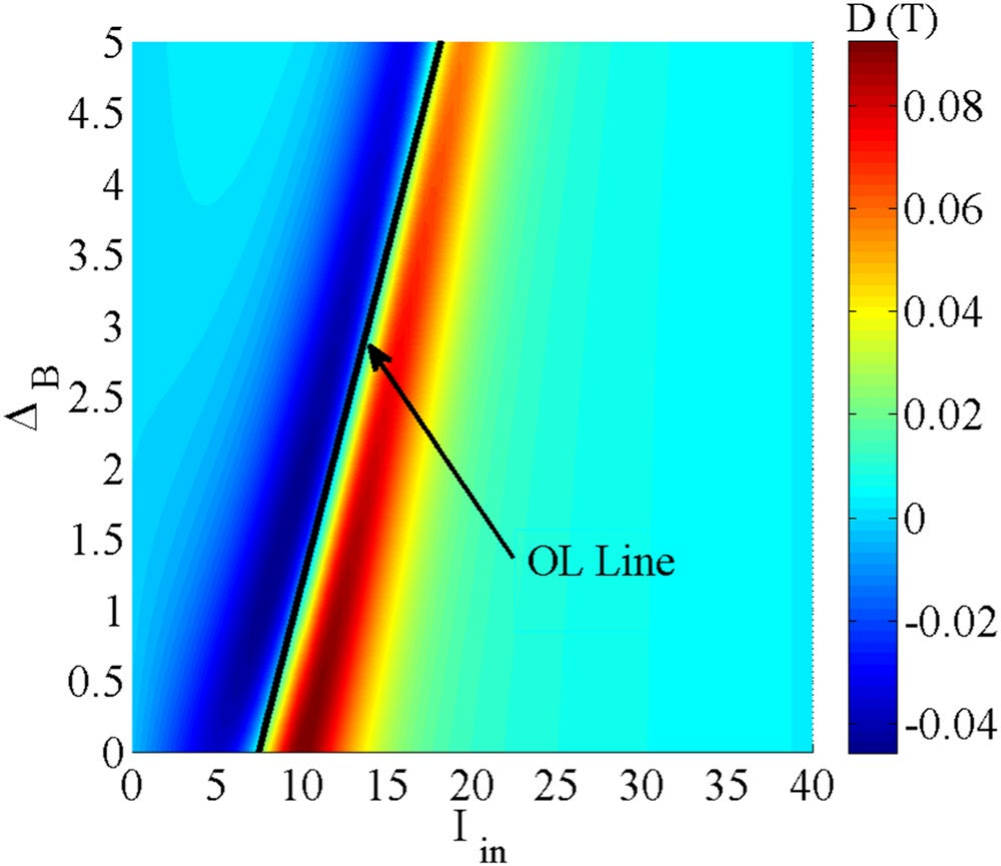
Slope of the transmission of the probe field versus the static magnetic field and intensity of the input probe field. The used parameters are Ω_*p*_ = 0.01*γ*, Ω_*c*_ = 65*γ*, Δ_*c*_ = 100*γ*, Δ_*p*_ = 1.5*γ*, Δ_*s*_ = 0, Ω_*s*_ = 2*γ* and *αl* = 800*γ*.

In order to gain a deeper insight, the slope of transmission of the probe field, D(T), is displayed in Fig. 7 as a function of the intensity of the input probe field and the static magnetic field. The used parameters are Ω_*p*_ = 0.01*γ*, Ω_*c*_ = 65*γ*, Δ_*c*_ = 100*γ*, Δ_*p*_ = 1.5*γ*, Δ_*s*_ = 0, Ω_*s*_ = 2*γ* and *αl* = 800*γ*. It is worth to note that for the OL range, the slope of the transmission is zero and even negative. Otherwise, the slope of the transmission is positive for the SA region. Figure 7 delineates the behavior of the RSA and the corresponding OL induced in the system as well as the SA for all values of the static magnetic field and intensity of the input field. This figure helps us to determine the OL range needed for different optical devices by selecting the appropriate parameters. The OL line, shown in the Fig. 7, presents the zero slope of transmission at the end of the RSA region. The left side of the OL line specifies the OL range, while the right side determines the SA region.

### Z-scan technique

**Figure 8:**
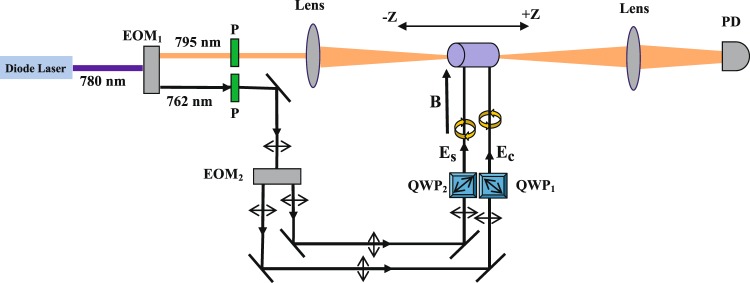
Schematic of experimental setup including the open aperture Z-scan technique. EOM is an electro-optical-modulator; P is a high-quality polarizer; QWP is a quarter wave plate; and PD is a photodiode.

In Fig. 8, the schematic of experimental setup is displayed including the open aperture Z-scan technique. A diode laser at 780 nm passes through the Electro-Optic Modulator (EOM) to generate a probe field at 795 mm and coupling field at 762 nm. The generated fields then pass through a high-quality polarizer (P1) to have linear polarization. The probe field is sent to organize the open aperture Z-scan part of the setup. The coupling field passes through the EOM2 to generate the two linearly polarized coupling fields. These two fields then pass through the quarter wave plate 1 (QWP_1_) and 2 (QWP_2_) to form the right- (E_*c*_) and left- (E_*s*_) circularly polarized coupling field, respectively, and are applied to the medium. In addition, a static magnetic field is applied to the medium parallel to the coupling fields. The sample is moved around the focal point of the focused probe field by a fine micropositioner. Finally the transmission of the probe field in each step is detected by the PhotoDiode (PD).

**Figure 9:**
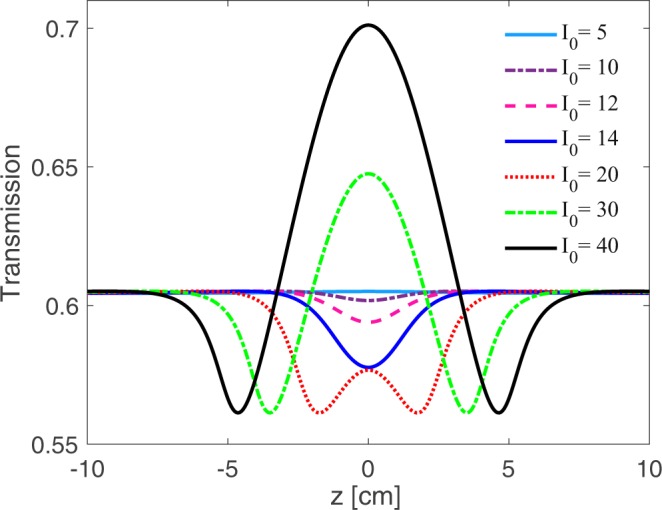
Z-scan measurements of the obtained OL using input gaussian probe field at 800 nm wavelength for different values of input intensity.

Finally, we employ the Z-scan technique to confirm the validity of the obtained theoretical results. The dip in the Z-scan transmission curve corresponds to the OL, while the peak stands for the SA effect. In Fig. 9, Z-scan technique measurement is displayed to investigate the z-dependent transmission for different values of input intensity chosen from the RSA and the SA region in Figs. 3 and 5. In Fig. 3, it was observed that the RSA was induced in the system up to a certain intensity called OL threshold. After the OL threshold, the SA is dominant in the system. These results was followed by the Fig. 5 in which it was observed that in the RSA region (*I*_*in*_ < 15*γ*), limited by the OL threshold, the OL appears. These results are demonstrated in the Z-scan technique presented in Fig. 9. The parameters that their results are examined in Fig. 9 are Ω_*p*_ = 0.01*γ*, Ω_*c*_ = 65*γ*, Δ_*c*_ = 100*γ*, Δ_*p*_ = 1.5*γ*, Δ_*s*_ = 0, Ω_*s*_ = 2*γ*, Δ_*B*_ = 3*γ* and *αl* = 800*γ*. It is seen in Fig. 9 that as the sample approaches and then moves away from the focal point, which its intensity is chosen from the RSA region in Figs. 3 and 5 (*I*_*in*_ < 15*γ*), the transmission curve takes the form of a dip. On the contrary, when the intensity of the focal point is chosen from the SA region in Figs. 3 and 5 (*I*_*in*_ > 15*γ*), the z-scan curve looks like a peak. Thus, the theoretical Z-scan experiment results are in good agreement with the results mentioned in Figs. 2–6.

## Conclusion

In summary, coherent generation and control of the RSA and OL are reported in a four-level Y-type quantum system. It was shown that the RSA is coherently induced by applied laser fields. We showed that, consequently, the OL is coherently induced through the RSA region so that all characteristics of the induced OL such as the intensity range and the threshold intensity can be controlled by either intensity or frequency of the laser fields. In addition, we proved that the static magnetic field has a constructive role in extending the RSA region and the OL range. Besides, it was demonstrated that the transmission of the suggested optical limiter can be controlled by either increasing the length of the medium or getting the atomic medium denser. Finally the obtained theoretical results was confirmed by the Z-scan technique. Our presented scheme can be used in designing the optical limiters with controllable intensity range and OL threshold.
